# Palliative Care for Patients on Extracorporeal Membrane Oxygenation for COVID-19 Infection

**DOI:** 10.1177/10499091211001009

**Published:** 2021-03-09

**Authors:** Anirudh Rao, Akram M. Zaaqoq, In Guk Kang, Erin M. Vaughan, Jose Flores, Victor J. Avila-Quintero, Muhtadi H. Alnababteh, Anne M. Kelemen, Hunter Groninger

**Affiliations:** 1 Department of Medicine, 12230 Georgetown University School of Medicine, Washington, DC, USA; 2 Department of Medicine, Section of Palliative Care, 8405 MedStar Washington Hospital Center, Washington, DC, USA; 3 Department of Critical Care Medicine, 8405 MedStar Washington Hospital Center, Washington, DC, USA; 4 12228 Yale University School of Medicine, New Haven, CT, USA

**Keywords:** extracorporeal membrane oxygenation, COVID-19, palliative care, acute respiratory distress syndrome

## Abstract

**Background:**

Critically ill patients with COVID-19 infection on extracorporeal membrane oxygenation (ECMO) face high morbidity and mortality. Palliative care consultation may benefit these patients and their families. Prior to the pandemic, our institution implemented a policy of automatic palliative care consultation for all patients on ECMO due to the high mortality, medical complexity, and psychosocial distress associated with these cases.

**Objectives:**

The main objective was to describe the role of the palliative care team for patients on ECMO for COVID-19 infection. The secondary objective was to describe the clinical outcomes for this cohort.

**Design:**

Case series.

**Settings/Subjects:**

All patients age 18 or older infected by the novel coronavirus who required cannulation on ECMO from March through July of 2020, at an urban, academic medical center in the United States. Inter-disciplinary palliative care consultation occurred for all patients.

**Results:**

Twenty-three patients (median age 43 years [range 28-64], mean body mass index 34.9 kg/m2 [SD 9.2], 65% Hispanic ethnicity) were cannulated on ECMO. Eleven patients died during the hospitalization (48%). Patients older than 50 years of age demonstrated a trend toward increased odds of death compared to those younger than 50 years of age (OR 9.1, *P* = 0.07). Patients received an average of 6.8 (SD 3.7) palliative clinical encounters across all disciplines. The actions provided by the palliative care team included psychosocial support and counseling, determination of surrogate decision maker (for 100% of patients), pain management (83%), and non-pain symptom management (83%).

**Conclusions:**

Here, we present one of the first studies describing the patient characteristics, outcomes, and palliative care actions for critically ill patients with COVID-19 on ECMO. Almost half of the patients in this cohort died during their hospitalization. Given the high morbidity and mortality of this condition, we recommend involvement of palliative care for patients/families with COVID-19 infection who are on ECMO. The impact of palliative care on patient and family outcomes, such as symptom control, satisfaction with communication, rates of anxiety, and grief experience merits further investigation.

## Introduction

The novel severe acute respiratory syndrome coronavirus 2 (SARS-CoV-2), which causes the coronavirus disease 2019 (COVID-19) infection, has infected over 27 million individuals in the United States, and has resulted in over 470,000 deaths.^1^ COVID-19 infection can range in severity, from asymptomatic carriage to respiratory failure requiring hospitalization, and in some cases, progressing to multi-organ system failure and death.^2-4^ Treatment options for this novel infection, especially when causing its most severe manifestations, are still limited.^5^ Efforts to rapidly develop a vaccine have succeeded in bringing several vaccines to market for distribution in record time.^6^

Patients who develop acute respiratory distress syndrome (ARDS) secondary to COVID-19 infection face high rates of morbidity and mortality.^7^ Supportive interventions for ARDS include mechanical ventilation, prone positioning, neuromuscular blockade, and extracorporeal membrane oxygenation (ECMO).^8,9^ Two randomized controlled trials have confirmed the safety and the potential effectiveness of ECMO in severe ARDS.^10,11^ During the H1N1 influenza pandemic in 2009, outcomes for patients requiring ECMO for viral infection-associated acute lung injury were favorable.^12-14^ However, studies on long-term outcomes of patients requiring ECMO are limited.^15^ The early observational reports from China on the use of ECMO for ARDS secondary to COVID-19 infection demonstrated a high mortality rate of 94%.^16,17^ By contrast, a more recent cohort from France reported a mortality of 31%, which was similar to previously reported studies on ECMO for severe ARDS,^18^ and a cohort from New York demonstrated 96% survival, to date.^19^

Specialist palliative care teams play an essential role in the care of patients with COVID-19 infection.^20-23^ Specific palliative care team actions can include symptom management, assistance with complex medical decision making, and patient/family support including spiritual, psychological, and bereavement care.^24,25^ The need for these actions is particularly evident when patients are critically ill and require advanced medical technologies such as ECMO, arguing for the involvement of palliative care in these situations.

The main objective of this study was to describe role of the palliative care team for patients on ECMO for COVID-19 infection. The secondary objective was to describe the clinical outcomes for this cohort.

## Methods

### Study Design

With institutional review board approval, we retrospectively analyzed the hospital course of all patients with confirmed COVID-19 infection on ECMO. The study period was from March 27 through July 31, 2020. All patients included had laboratory-confirmed SARS-CoV-2 infection, as detected by reverse-transcriptase polymerase chain reaction of specimens from nasopharyngeal swabs, and were 18 years of age or older. Patients cannulated on both venovenous-ECMO (VV-ECMO) and venoarterial-ECMO (VA-ECMO) were included. Investigators extracted data from the electronic health record (EHR) regarding patient baseline characteristics, clinical interventions implemented, and outcomes at hospital discharge. Complete data were available for all patients at the time of data analysis. Patients who were considered for ECMO but were not ultimately cannulated were excluded from this study. None of the patients in this study received palliative care consultation prior to ECMO cannulation. Demographic information extracted included age, race, ethnicity, sex, body mass index (BMI), and comorbidities. Measures of palliative care team interactions with the patient and/or family, including the discipline of the team members who were involved, were also extracted from the EHR. Palliative care “encounters” were defined as actions performed directly with the patient and/or family. “Family meetings” were defined as scheduled meetings with members of the patient’s family, the palliative care team and the intensive care unit (ICU) team. When indicated, all patient and family encounters were conducted with the use of in-person or telephonic interpreter services per hospital policies.

### Setting

MedStar Washington Hospital Center is a 912-bed academic tertiary care center in Washington, DC, and is a high-volume ECMO center, with an average of 80 patients cannulated on ECMO each year. Prior to the pandemic, in January 2020, clinical leaders in cardiac surgery, surgical critical care, and palliative care agreed to automatic palliative consultation for any hospitalized patient receiving ECMO within 48 hours of cannulation. For each case, the specific consultation request (for example, symptom management, advance care planning including interpretation of advance directives, complex medical decision making, psychosocial-spiritual support to patient/family, etc.) was determined collaboratively between stakeholder teams. At our institution, interdisciplinary palliative care consultation may include medical, psychosocial, chaplain, and pharmacy assessments. Prior to the pandemic, the majority of patients requiring ECMO were on VA-ECMO for hemodynamic support following cardiac surgery. By protocol, the palliative care team was notified by the critical care team when patients were cannulated on ECMO.

### Data Analysis

Descriptive statistics were used to report patient characteristics, clinical course, and palliative care services received. Patient characteristics were expressed as N (%) for categorical variables, mean (standard deviation, SD) for continuous variables, and median (range; inter-quartile range), as appropriate. *P*-values for continuous variables correspond to 2 Sample T-Test. Logistic regressions were used to identify demographic predictors and comorbidities to estimate the odds of death.

Time-to-death from ECMO cannulation was estimated based on the date of ECMO cannulation (as the origin) and date of death (as the event of interest for time-to-event analyses) or date of discharge (as right censoring). Descriptive statistics about selected time-to-event outcomes are described in the text of the results. In addition, Kaplan-Meier curves were used to visualize differences in the time-to-death from ECMO cannulation to death, comparing patients who were younger than 50 years to those who were older than 50 years. Parametric regression (accelerated-failure-time models) with Weibull distributions were used to estimate Time Ratios (TRs). TRs are an alternative and effective measure of Hazard ratios.^26^ TRs >1 correspond with prolongation of time-to-death from ECMO cannulation while TRs <1 signify a shortened time-to-death from ECMO cannulation. For all analyses, the Type-I error rate to evaluate statistical significance was set at 0.05. Data management and analyses were performed with STATA/IC statistical software, version 16 (StataCorp LLC).

## Results

Twenty-three patients were included in the analysis; patient characteristics and comorbidities are listed in Table 1. Our population was predominantly of Hispanic ethnicity (65%), obese (mean BMI 34.9 kg/m2), and young (median age 43 years). The median hospital length of stay was 28 days (range 4-82; IQR 24-35). 22/23 patients (96%) were cannulated with VV-ECMO for acute hypoxic respiratory failure, and one patient was cannulated on VA-ECMO for COVID-19 associated myocarditis. 11/23 patients died during the hospitalization, indicating a mortality rate of 48%.

**Table 1.** Patient Characteristics (N = 23).

**Characteristic**	***N* = 23**
*Sex, n (%)*	
Male	15 (65.2%)
Female	8 (34.8%)
*Age, median (range; IQR)*	43 (28-64; 37-50)
*BMI, mean (SD)*	34.9 (9.2)
*Race, n (%)*	
African American	7 (30.4%)
Asian	1 (4.4%)
White	0 (0%)
*Ethnicity, n (%)*	
Hispanic	15 (65.2%)
Non-Hispanic	8 (34.9%)
*Comorbidities, n (%)*	
Hypertension	5 (21.7%)
COPD/Asthma	4 (17.4%)
Diabetes Mellitus	7 (30.4%)
Smoking	3 (13.0%)
Obesity^a^	13 (56.5%)
Pregnant or Peripartum	2 (8.7%)

Abbreviations: BMI, body mass index (calculated as weight in kilograms divided by height in meters squared). COPD, chronic obstructive pulmonary disease. IQR, inter-quartile range. ^a^ Obesity is defined as BMI greater than 30.

### Patient Outcomes

Twelve patients survived to hospital discharge (52%). 5/12 patients (42%) were discharged to their respective residences (4 patients to home, 1 to a correctional facility), 4/12 patients (33%) were discharged to a rehabilitation facility, and 3/12 patients (25%) were transferred to an acute care hospital outside our hospital network either for consideration of lung transplant (n = 2) or due to insurance reasons (n = 1).

**Figure 1.**
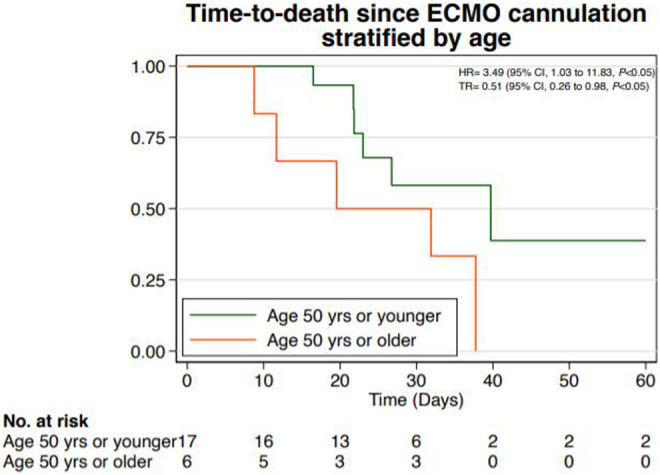
Time-to-event analysis from date of ECMO cannulation to date of death, stratified by age greater than or less than 50 years. The time ratio (TR) demonstrates a significant reduction in time-to-death for the group older than 50 years of age, compared to the group of patients age 50 years or younger (*P* < 0.05). Hazard ratio (HR) displayed in figure.

Eleven patients died during the hospitalization, indicating a mortality rate of 48%. Of the patients who died, 9/11 patients (82%) died following a change in goals of care toward withdrawal of life-sustaining medical therapies (LSMT) and allowing natural death. One patient received cardiopulmonary resuscitation (CPR) and advanced cardiac life support (ACLS) measures at the time of death, whereas 10/11 patients (91%) had a change in code status to “No CPR” prior to their death. Of the patients who died, 4/11 patients (36%) received CPR at some point during their hospitalization, and 3/11 patients (27%) received tracheostomy placement. Based on goals of care discussions with surrogates resulting in a change in treatment preferences toward comfort-focused care, 7/23 (30%) patients died following withdrawal of ECMO therapy. Of those who were able to be decannulated from ECMO based on clinical parameters, 4 patients died after decannulation. In the entire cohort, all patients who were age 50 or older (n = 6) died. Using logistical regressions, there were no statistically significant associations between any of the demographic variables or comorbidities on the odds of death. Comparing patients older than 50 years of age to those younger than 50 years demonstrated a trend toward increased odds of death for the older patients (P = 0.07) (Table 2). Moreover, the median time-to-death from ECMO cannulation among patients older than 50 years of age was approximately half as long as compared to patients 50 years or younger (TR = 0.51, 95% CI 0.26 to 0.98, P < .05), as seen in Figure 1.

**Table 2.** Logistic Regressions for Demographic Predictors on the Odds of Death, With Odds Ratio (OR), 95% Confidence Intervals (CI), and *P* values.

	OR	95% CI	*P* value
Age^1^	1.07	0.97 to 1.19	0.16
Age 50 years or older vs younger	9.17	0.86 to 97.69	0.07
Male vs. Female	4.50	0.67 to 30.23	0.12
Non-Hispanic vs. Hispanic	1.14	0.21 to 6.37	0.88
Obese	0.29	0.05 to 1.59	0.15
Hypertension	1.88	0.25 to 14.08	0.54
COPD	1.11	0.13 to 9.61	0.92
Diabetes mellitus	0.75	0.13 to 4.49	0.75
Active smoker	2.44	0.19 to 31.53	0.49
Malignancy	1.10	0.06 to 20.01	0.95

^1^Odds of death for each additional year of age.

For patients who were able to be decannulated from ECMO, the median days on ECMO was 11 (range 3-20; IQR 10-15). Nearly all patients in this study (N = 22, 96%) were intubated during the hospitalization, prior to ECMO cannulation. The one patient who did not require intubation had cardiogenic shock from COVID-19 associated myocarditis. The 6 patients who were successfully extubated without need for tracheostomy and long-term ventilatory support were intubated for a median of 18 days (range 7-25; IQR 14-22). Of the 4 patients who required tracheostomy, one patient was transferred to an outside hospital, one patient was able to have the tracheostomy decannulated prior to hospital discharge, and 2 patients were transferred to a long-term ventilator-weaning facility.

Nine patients (9/23; 39%) in the cohort required renal replacement therapy (RRT) during their hospitalization. Of these, 6/9 patients died and 3/9 survived to hospital discharge. The 3 patients who survived were discharged to rehabilitation facilities. One patient required chronic hemodialysis whereas the other 2 patients experienced renal recovery. The need for RRT was not significantly associated with increased odds of death (OR 3.6, 95% CI 0.61 to 21, *P* = 0.16).

### Palliative Care Actions

Palliative care consultation occurred for all patients within 48 hours of ECMO cannulation, per our institutional ECMO clinical care process described earlier. None of the patients in this study received palliative care consultation prior to ECMO cannulation. Patients and/or their families received an average of 6.8 (SD 3.7) palliative clinical encounters, composed of visits by a physician or nurse practitioner (average 2.6 visits, SD 1.8), a palliative social worker (average 3.9 visits, SD 1.8), or a palliative chaplain (average 1.6 visits, SD 2.4). Palliative clinicians participated in an average of 1.0 family meetings (SD 1.6) per case (Table 3). Due to restrictions on visitation, clinical encounters with family members usually occurred by telephone and family meetings occurred over teleconferencing software. Spanish interpreter services were required for 14/23 (61%) patients/families and were included in the telephone or teleconference encounters.

**Table 3.** Palliative Care Actions With Comparison of Mean Number of Actions Received for the Group That Survived and the Group That Died, Analyzed With Two Sample T-Test.

Palliative Care Action	Patients who received the action at least once N (%)	Mean number of the action for patients who survived N = 12. Mean (SD)	Mean number of actions for patients who died N = 11. Mean (SD)	*P*-value
Determine surrogate decision maker	23 (100)	1.00 (0.00)	1.09 (0.30)	0.31
Disease state education	11 (48)	0.58 (0.79)	1.18 (1.60)	0.26
Pain management	19 (83)	1.08 (0.67)	1.73 (1.35)	0.16
Non-pain symptom management	19 (83)	1.08 (0.67)	1.91 (1.58)	0.11
Clarify goals of care	11 (48)	0.67 (1.44)	1.45 (1.51)	0.21
Psychosocial support/counseling	23 (100)	3.00 (2.13)	3.09 (1.87)	0.91
Spiritual support/counseling	13 (57)	1.42 (2.81)	1.55 (1.69)	0.90
Determine treatment preferences	11 (48)	0.58 (0.67)	0.73 (1.01)	0.69
Family Meetings with palliative care team participation	9 (39)	0.75 (1.48)	1.18 (1.66)	0.52

All patients/families received psychosocial support and counseling and confirmation of surrogate decision maker from the palliative care team. None of the patients had completed an advance directive prior to the hospitalization. The next most common palliative care action was assistance with pain and non-pain (commonly dyspnea or delirium) symptom management, (n = 19, 83%). Typically, recommendations were provided to the ICU team following examination of intubated and sedated patients. There were no statistically significant differences between the mean number of palliative care actions for patients who ultimately survived to hospital discharge and those who did not, though there were trends toward increased involvement of the palliative care team for symptom management in the patients who died (Table 2). The action that was provided most frequently, regardless of the patient’s ultimate outcome, was psychosocial support and counseling. This mirrors the above finding illustrating that, among palliative care disciplines, social workers provided the most encounters to each patient/family.

## Discussion

To our knowledge, this is the first study describing the patient characteristics, outcomes, and role of palliative care for critically ill patients with COVID-19 on ECMO. In this case series of predominantly young, obese, Hispanic patients, we report a mortality rate of 48%. A recent study by Martinez et al. demonstrated that Hispanic patients in the Baltimore-Washington DC region were disproportionately affected by COVID-19 infection.^27^ Mirroring these findings, we report here that the majority of critically ill patients in this geographic region were Hispanic. These patients experienced protracted hospitalizations, with median LOS of almost 1 month, and frequent need for advanced medical interventions such as tracheostomy or RRT. Older patients (defined as age greater than 50 years) had poorer outcomes than younger patients, with a trend toward increased odds of death. From the date of ECMO cannulation, older patients died sooner than younger patients. Further analyses are required to better understand the reasons for this finding.

The palliative care team was heavily involved in the care of these patients and their families, with the most frequent actions being confirmation of healthcare decision maker, psychosocial support, and symptom management. The palliative care team’s actions were not statistically different between patients who ultimately survived to hospital discharge and those who did not. These findings suggest that the palliative care team provided broad-based palliative services regardless of the ultimate outcome.

We believe that involvement of palliative care for this critically ill population was of utility for not only the patients and family members, but also for the ICU staff. Previous studies have demonstrated the beneficial effects of palliative care in patients with conditions such as advanced cancer^28-31^ and advanced heart failure.^32,33^ A recent randomized controlled trial of early palliative care involvement in critically ill ICU patients resulted in increased use of hospice services, and a decrease in burdensome interventions at the end-of-life such as tracheostomies, without an increase in mortality compared to patients receiving usual care.^34^ The patients and families in our cohort received frequent psychosocial support and counseling from the interdisciplinary palliative care team. Additionally, early involvement of palliative care, during a time when the patient is critically ill but stabilized on ECMO, allowed the requisite time for rapport building with families and for determining surrogate decision-maker hierarchy. Early engagement also gave the palliative care team time to clarify the best practices for communication with each family unit by determining the mode of communication (telephone conference vs video conference) and the best way to incorporate interpreter services when indicated.^25^ Some of these functions served to lighten the burdens faced by the ICU staff, who were caring for up to 6 patients on ECMO at one point during a surge of COVID-19 cases. The benefits of an “embedded” palliative care team for hospital units caring for a high number of critically ill patients with COVID-19 may be even greater, as evidenced in one study of emergency department providers.^35^ Based on this evidence, we recommend involvement of palliative care for patients/families with COVID-19 infection who are on ECMO.

Specialist palliative care services remain a scarce resource in many hospitals across the country.^36^ Even at hospitals with robust palliative care teams, palliative care utilization in the time of the COVID-19 pandemic may be low,^37^ and possibly even more so for patients of racial and ethnic minorities.^38^ In order to improve access to palliative care services, studies have examined the use of web-based applications,^39^ tele-palliative care consultation,^40,41^ and work-force expansion through training and redeployment of psychiatry trainees.^42^ Early involvement at critical junction points, such as in the emergency department, seems effective to redirect goals of care such that patients avoid unwanted medical interventions.^43^ As the pandemic unfolds, it will be important for hospitals and health systems to allocate the necessary resources to provide palliative care for patients in need, in whatever form is most feasible.^44^

The limitations of this case series include the retrospective study design and small size of the cohort. The lack of a comparison group of patients who did not receive a palliative care consultation limits the ability to draw conclusions regarding the impact of the palliative care team on patient outcomes. The size of the cohort may have resulted in the failure to detect associations between pre-morbid clinical characteristics and the odds of death, or between the types and quantity of palliative care actions and ultimate outcome. Future studies are required to better understand the impact of palliative care team involvement in this critically ill patient population on family member and staff outcomes. The patient outcomes reported in this study may not be generalizable to other populations that have different racial and ethnic makeups. Additionally, our findings may not be generalizable to other hospitals without the staffing to implement palliative care consultation for all patients on ECMO.

## Conclusion

We present data from a case series of patients with severe COVID-19 infection on ECMO who received automatic palliative care consultation at a single, high-volume ECMO center. Palliative care was heavily involved in the care of these patients and their families, with most frequent actions being confirmation of healthcare decision maker, psychosocial support, and symptom management. In this population of mostly Hispanic, obese, young patients, the mortality rate was 48%. These patients experienced protracted hospitalizations with frequent need for advanced medical interventions, in addition to ECMO. Given the high morbidity and mortality, we recommend involvement of palliative care for patients/families with COVID-19 infection who are on ECMO. Further studies are needed to understand the impact of palliative care on patient and family outcomes, such as symptom control, satisfaction with communication, rates of anxiety, and grief experience.
